# Primary care in Ireland: history explains the system we see today

**DOI:** 10.1016/j.lanepe.2025.101300

**Published:** 2025-04-11

**Authors:** Domhnall McGlacken-Byrne

**Affiliations:** Royal College of Physicians in Ireland, Dublin, Ireland

Faulkner declared that the past is never dead—it's not even past.

Your March editorial focused on Ireland's unfair primary care system.^1^ Uniquely among our neighbours, we do not have universal healthcare and there is no basic entitlement to primary care.

To understand why, we must heed Faulkner's advice and look to the past. Words from history are imprinted across Ireland's health services to this day—like eligibility, sweepstakes, and subsidiarity.

Firstly, the culture of eligibility has deep roots, superseding values of solidarity and social democracy.^2^ In nineteenth century Ireland, those who could afford to pay a doctor, did so. Those who couldn't relied on the Poor Law dispensary and workhouse. When ill, one sought out a Poor Law Guardian—generally a local landowner or merchant. If deemed sufficiently destitute, one was issued a ticket, granting eligibility for dispensary care.

Formalised in 1838, this ticketing system was not formally replaced until 1953 and 1970, when Health Acts introduced medical cards.^2^ Yet strict eligibility categories persisted: if you had a medical card, GP care was free, but hard to access. If you didn't, GP care was faster, but expensive. This model endures today.

Secondly, consider the idiosyncrasy of the Irish Hospital Sweepstakes.^3^ While most democracies were constructing universal healthcare systems, through taxation or social insurance, Ireland relied on Sweepstakes raffles. These began in the 1930s as fundraisers for local hospitals. Returns exceeded all expectations; by 1931, Sweepstake raffles were providing £1 m per annum, at a time when all State expenditure on public assistance totalled £3 m.^3^ A massive, haphazard building spree embedded an over-reliance on small local hospitals at the expense of primary care.

Lastly, it may be surprising that an obscure papal encyclical from 1891 should leave an imprint on a health system—but it did. Subsidiarity was the Catholic doctrine delineating the relationship between Church and State: civil governments should intervene in matters like health and education in cases of “extreme necessity”, and only then.^3^ Such teachings were taken seriously in Ireland. The resultant arm's-length dynamic between governments and healthcare facilitated further fragmentation and decentralisation.

Ireland has made substantial progress in recent decades.^4^ However, for most of Irish history, healthcare was firmly situated in the realms of eligibility and charity. In the words of Fintan O'Toole, “we came as supplicants, not citizens”.^5^

This is the context in which Ireland became an outlier.

## Contributors

Domhnall McGlacken-Byrne conceptualised and wrote this letter.

## Declaration of interests

DMB—Chair, Doctors for Universal Healthcare.
